# Mechanical Modeling of Whisker-Filled Dispersed Isotactic Polypropylene: Matrix-Dominated Yielding and Fracture Mechanisms

**DOI:** 10.3390/polym18080917

**Published:** 2026-04-09

**Authors:** Tetsuo Takayama, Daisuke Shimizu

**Affiliations:** Graduate School of Organic Materials Science, Yamagata University, Yamagata 992-8510, Japan

**Keywords:** fracture toughness, isotactic polypropylene (iPP), mechanical modeling, shear yielding, whisker-like fillers

## Abstract

This study investigated mechanical properties of composite materials consisting of an isotactic polypropylene (iPP) matrix reinforced with whisker-like fillers: carbon nanofibers (CBNF) and wollastonite (WN). We strove to develop mechanical models specifically for predicting yield stress and fracture toughness. Experimentally obtained results validated findings obtained using the proposed models. Regarding the elastic modulus, data suggest that conventional rules of mixture, typically used for glass fiber-reinforced polymers, remain applicable, indicating that filler addition enhances stiffness in a predictable manner. However, yield stress and fracture toughness exhibited distinct behaviors. Results revealed that these properties are governed predominantly by shear yielding of the iPP matrix rather than reinforcement effect of the fillers. Despite the presence of whiskers, the overall yield and fracture mechanisms depend heavily on the matrix’s plastic deformation and energy dissipation. The constructed models consistently explain these findings, supporting quantitative evaluation of the matrix’s contribution. These results emphasize that developing high-performance iPP composites requires knowledge of the intrinsic ductile properties of the matrix alongside filler selection and dispersion.

## 1. Introduction

Polypropylene (PP), a premier commodity thermoplastic, is known for its balanced properties and cost-efficiency [1]. Among its forms, isotactic polypropylene (iPP) is particularly important because of its stereoregularity, which leads to high crystallinity and superior mechanical strength, chemical resistance, and heat stability [1,2]. Consequently, iPP is indispensable across the automotive, appliance, and medical sectors [1]. However, in contrast to engineering plastics or metals, iPP requires enhancement of stiffness and impact resistance to meet the demands associated with structural applications.

Fiber reinforcement is the most effective strategy for overcoming these limitations [3]. Traditionally, glass fiber (GF) has been the dominant filler because of its excellent cost–performance ratio in reinforcing iPP [4,5]. Yet, the transition toward a circular economy has highlighted the poor mechanical recyclability of glass fiber reinforced plastics (GFRP) [6]. During recycling, high shear forces cause considerable fiber breakage. When the fiber length falls below the “critical fiber length,” the aspect ratio diminishes, leading to a sharp decline in mechanical properties and necessitating “downcycling” [7].

Whisker-like fillers offer a promising alternative to resolve this dilemma. Whiskers, which are fine, single-crystalline needles with nearly perfect structures, exhibit mechanical strengths that approach theoretical limits [8]. Examples include silicon carbide, carbon nanofibers (CBNF), and naturally derived minerals such as wollastonite (WN) [9,10,11,12,13,14]. In fact, whiskers possess several advantages over GF: superior mechanical properties per unit weight, submicrometer diameters that enhance surface smoothness, and potentially better recyclability. Because whiskers are inherently fine, they might maintain an effective aspect ratio even after experiencing shear forces during recycling processes [15].

Although whisker reinforcement is well known to enhance stiffness (elastic modulus) [16,17], its influence on inelastic behavior, such as yield stress and fracture toughness, remains inadequately understood. Mechanical modeling is necessary to bridge the gap separating microscopic structural parameters, such as filler volume fraction, aspect ratio, and interfacial adhesion, and macroscopic properties [18,19]. Whereas elastic modulus can often be predicted by the rule of mixtures or Halpin–Tsai equations [20,21], these conventional models often fail to account for yield stress and fracture toughness because of complex local energy dissipation mechanisms, such as shear yielding and crack propagation [22,23].

Existing models, such as the Kelly–Tyson and Pukanszky models, were developed primarily for rigid GF systems [22,23,24,25]. It remains unclear if these apply to nanoscale whisker fillers in a ductile iPP matrix. Specifically, when fillers are extremely fine, their overall behavior might be governed more by matrix deformation (e.g., shear yield band formation) than by the filler’s own fracture or pull-out.

This study investigates iPP composites reinforced with two distinct whisker-like fillers: CBNF and WN. By experimentation, we evaluated their elastic modulus, yield stress, and fracture toughness. The core objective is to construct mechanical models that distinguish direct filler reinforcement from the filler’s influence on the shear yielding behavior of the iPP matrix. This research was conducted to provide a design framework for high-performance, recyclable polymer composites by clarifying the governing factors of their mechanical response.

## 2. Materials and Methods

### 2.1. Materials

The materials used for this study are shown in Table 1. Isotactic polypropylene (iPP, Novatec PP MA1B; Japan Polypropylene Corp., Tokyo, Japan) was used as the matrix. iPP was selected because it is a representative polymer material among general-purpose plastics and, as a crystalline polymer, exhibits distinct yield behavior (shear yield); consequently, it was chosen as a model material suitable for verifying the mechanical modeling in the inelastic region, which is the objective of this study. Maleic anhydride-modified polypropylene (MAHPP, SCONA TSPP 10213 GB; BYK Additives & Instruments Co., Ltd., Wesel, Germany) was used as a coupling agent to improve interfacial adhesion between the fillers and the matrix.

Fillers of three types were used: carbon nanofibers (CBNF, ALP-NA1; Almedio Inc., Tokyo, Japan), wollastonite (WN, WFB5; Nippon Talc Co., Ltd., Osaka, Japan), and glass fibers (GF, T-351; Nippon Electric Glass Co., Ltd., Shiga, Japan). The CBNF had 0.2–0.8 µm fiber diameter and 1–15 µm length. The WN had 5–6 µm fiber diameter and 60–72 µm average fiber length. Before compounding, the GF had 13 µm fiber diameter and 3 mm initial fiber length. CBNF was selected as synthetic whiskers, WN as natural whiskers, and GF as a filler for comparison with the whiskers.

### 2.2. Fabrication of Composites via Melt Compounding

The materials were melt-compounded using a twin-screw extruder (15 mm screw diameter, L/D, 24IMC0-00; Imoto Machinery Co., Ltd., Kyoto, Japan). Compounding was performed at a 230 °C melt temperature and a 75 rpm screw rotation speed. The filler content for each composite was adjusted as follows: 5, 10, 15, 20, and 30 wt% for CBNF; 5, 10, and 20 wt% for WN; and 5, 10, 20, and 30 wt% for GF. The ranges for the CBNF and WN content were selected as the limits within which the reinforcing effect of the fillers could be clearly confirmed while maintaining good processability (flowability) during melt mixing and injection molding. The GF content was selected to correspond with the CBNF and WN content levels.

### 2.3. Preparation of Test Specimens via Injection Molding

Pellets obtained from melt compounding were injection-molded using a micro-electric injection molding machine (10 mm plunger diameter, 29.7 kN clamping force, C,Mobile0813; Shinko Sellbic Co., Ltd., Tokyo, Japan) to prepare test specimens. The injection temperature was set as 230 °C. The mold temperature was set at 50 °C and 80 °C. Detailed molding conditions are presented in Table 2.

**Figure 1:**
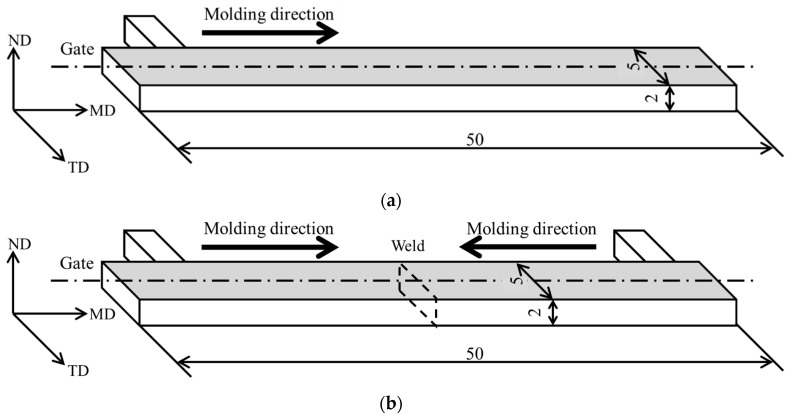
Specimen geometries of injection products: (**a**) single-gate and (**b**) double-gate. These figures were obtained from an earlier study [16]. In the figure, TD, WD, and MD, respectively, denote the thickness direction (TD), width direction (WD), and molding direction (MD).

The molded part shape is shown in Figure 1. The molded specimens were rectangular bars: 50 mm long, 5 mm wide, and 2 mm thick. Specimens were fabricated with either a single gate (no weld line) or two gates (with a weld line).

### 2.4. Density Measurement

The density of the fabricated composites was measured using an electronic densimeter (MDS-300; Alfa Mirage Co., Ltd., Osaka, Japan). Measurements were taken according to the Archimedes (buoyancy) method. Using the experimentally obtained results, the filler density was estimated according to the following rule of mixtures, as Equation (1) [26].

(1) $$\rho_{f}=\frac{\rho_{c}-\rho_{m}(1-V_{f})}{V_{f}}$$

Here, ρ represents the density. V denotes the volume fraction. Subscripts m, f, and c, respectively, signify the matrix, filler, and composite.

### 2.5. Melt Volume–Flow Rate (MVR) Measurement

To evaluate the melt flowability of the composites, the melt volume–flow rate (MVR) was measured using a melt flow indexer (G-01; Toyo Seiki Seisaku-sho, Ltd., Tokyo, Japan). Measurements were conducted in accordance with ISO 1133 at 230 °C and with a load of 2.160 kgf [27]. The test was performed once per condition because the equipment which was used captures MVR data in real time every second, allowing for a reliable average value to be obtained from a single test.

### 2.6. Three-Point Bending Test

**Figure 2:**
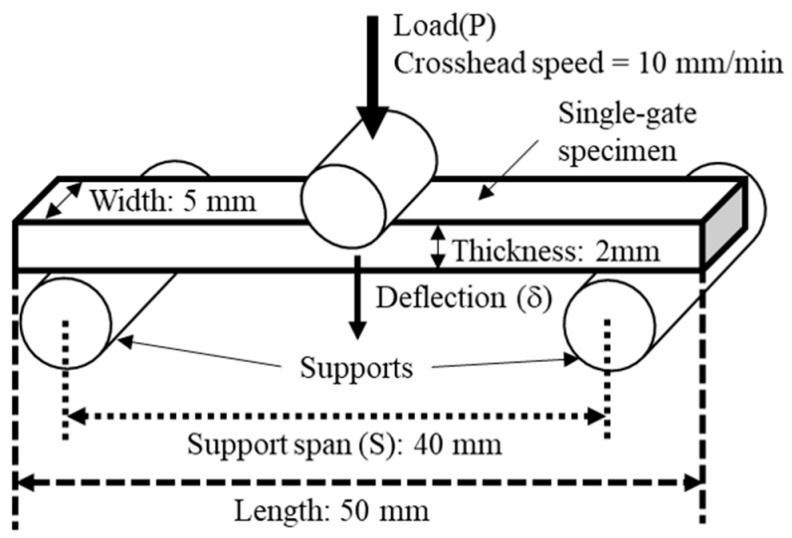
Schematic diagram of 3-point bending test.

To evaluate the flexural properties of the composites, three-point bending tests were performed using a small-scale universal mechanical tester (MCT-2150; A&D Co., Ltd., Tokyo, Japan) in accordance with ISO 178 [28]. A schematic diagram of this test is shown in Figure 2. Single-gate specimens were positioned such that the load was applied in the width direction (WD). Tests were conducted with a 40 mm support span and 10 mm/min crosshead speed to obtain load–deflection curves.

Flexural stress and flexural strain were calculated from the load and deflection data to generate stress–strain curves. The formula for flexural stress (2) is presented below.

(2) $$\sigma_{f}=\frac{3PS}{2bh^{2}}$$

Therein, P stands for the load, S denotes the span, b represents the width of the test specimen, and h expresses the test specimen thickness. The formula for flexural strain (3) is presented below.

(3) $$\varepsilon_{f}=\frac{6\delta h}{S^{2}}$$

In that equation, δ represents deflection. The maximum flexural stress on this curve was defined as the flexural strength. The slope of the initial linear region was calculated as the flexural modulus.

Furthermore, assuming the entire cross-section of the specimen reached yielding, the flexural yield initiation stress was defined as 2/3 of the flexural strength. The flexural strain at this point was defined as the flexural yield initiation strain. Based on the method proposed by Takayama et al. [29], Poisson’s ratio at yield initiation was calculated using Equation (4).

(4) $$\upsilon =\frac{\sigma_{fy}}{3E_{f}\varepsilon_{fy}-\sigma_{fy}}$$

Here, σ_fy_ stands for the flexural yield stress, E_f_ represents the flexural modulus, and ε_fy_ denotes the flexural yield strain. Additionally, assuming that deformation during bending was pure extensional flow, the yield initiation stress was found using Equation (5).

(5) $$\sigma_{y}=\frac{\sigma_{fy}}{1+\upsilon }$$

Five tests were conducted for each condition. The average values were taken as the characteristic properties. The standard deviation was calculated as an indicator of variation.

### 2.7. Short-Beam Shear Test

**Figure 3:**
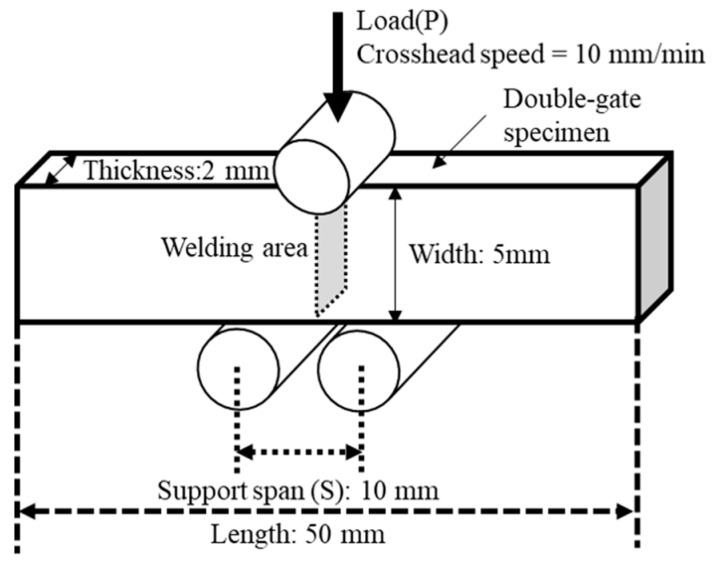
Schematic diagram of short beam shear test.

A short-beam shear test was conducted to evaluate the interfacial adhesion between the filler and the matrix. A schematic diagram of this test is shown in Figure 3. Using a universal mechanical tester (MCT-2150; A&D Co., Ltd., Tokyo, Japan), two-gate specimens were positioned in the width direction (WD) such that the load was applied to the weld line. The support span was set as 10 mm. The crosshead speed was 10 mm/min.

Evaluation was performed according to the method proposed by Jiang et al. [30]. First, a stiffness–load curve, representing the change in specimen stiffness, was obtained by differentiating the load–deflection curve with respect to deflection. From this curve, two points at which the stiffness decreased discontinuously were extracted. The shear stress at each point was calculated using the following equation.

(6) $$\tau =\frac{3P}{4bh}$$

Finally, the interfacial shear strength (IFSS) was calculated by combining the two shear stress values according to the following equation.

(7) $$\tau_{I}=\sqrt{\tau_{1}^{2}+\tau_{2}^{2}}$$

Therein, subscripts 1 and 2 were assigned starting from the lower shear stress. Five tests were conducted for each condition to ascertain the mean and standard deviation.

### 2.8. Notched Charpy Impact Test

**Figure 4:**
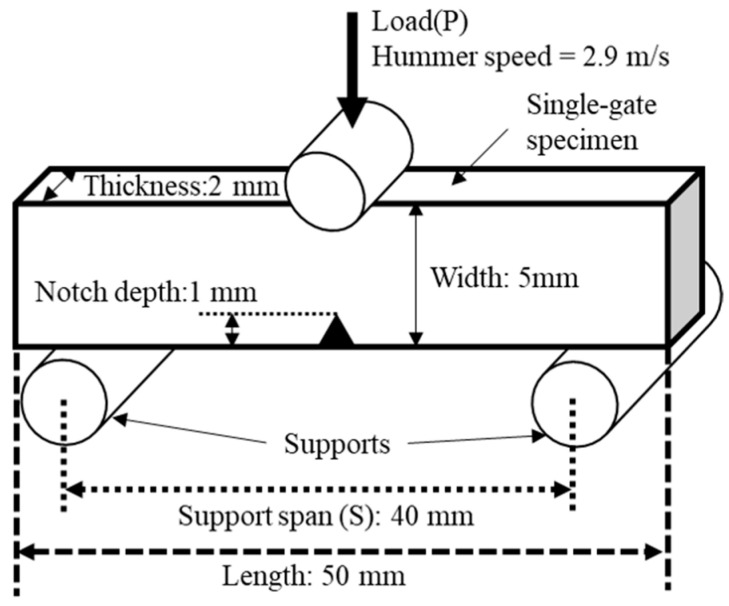
Schematic diagram of notched Charpy impact test.

To evaluate the impact resistance of the composites, notched Charpy impact tests were performed in accordance with ISO 179 using a Charpy impact tester (Maise Test Equipment Co., Ltd., Kyoto, Japan) [31]. A schematic diagram of this test is shown in Figure 4. A V-notch with 1 mm depth was introduced into the center of the specimen in the width direction (WD) via machining. Tests were conducted with a 40 mm support span and 2.91 m/s impact velocity.

### 2.9. Observation of Fracture Surfaces

To investigate details of the impact fracture behavior and interfacial states, the fracture surfaces of the specimens after the notched Charpy impact tests were observed using a scanning electron microscope (SEM, TinySEM-510; Technex Lab Co., Ltd., Tokyo, Japan). Observations primarily emphasized the region near the notch tip.

## 3. Results

### 3.1. Composite Density and Filler Density

**Figure 5:**
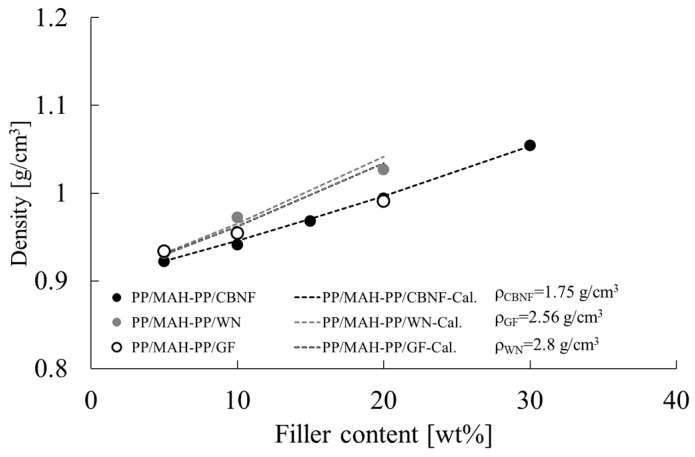
Density of polypropylene/maleic anhydride-grafted polypropylene (PP/MAH-PP) composites as a function of filler content for carbon nanofiber (CBNF), wollastonite (WN), and glass fiber (GF). The plots present experimentally obtained values, whereas the dashed lines (-Cal.) show theoretical values calculated based on the respective filler densities: ρ_CBNF_ = 1.75 g/cm^3^, ρ_GF_ = 2.56 g/cm^3^, and ρ_WN_ = 2.8 g/cm^3^.

When evaluating the mechanical properties of composite materials, weight (density) is an important factor, particularly for application to transportation equipment. Therefore, the density of each fabricated composite was measured to elucidate changes relative to filler loading (wt%). Figure 5 portrays the experimentally measured densities (plotted points) alongside the theoretical densities (dashed lines). The theoretical values were calculated using the matrix density (approximately 0.91 g/cm^3^) and the estimated density for each filler (ρ_CBNF_ = 1.75 g/cm^3^, ρ_GF_ = 2.56 g/cm^3^, ρ_WN_ = 2.8 g/cm^3^), which was chosen based on the rule of mixtures to explain the experimentally obtained results best. It has been reported that there is virtually no difference in density between iPP and MAH-PP; therefore, in this study, we calculated the theoretical values based on the assumption that the change in density due to the MAH-PP content is extremely small [32].

In all samples, the composite density increased linearly with the increase in filler loading. The experimentally measured values showed excellent agreement with the theoretical calculation lines (dashed lines) which were inferred using the previously described estimated parameters. Comparison by filler type at the same loading (e.g., 20 wt%) revealed that PP/MAH-PP/WN showed the highest density (approx. 1.02 g/cm^3^), followed by PP/MAH-PP/GF (approx. 1.00 g/cm^3^), and then PP/MAH-PP/CBNF (approx. 0.98 g/cm^3^).

### 3.2. Melt Flowability of Composites

**Figure 6:**
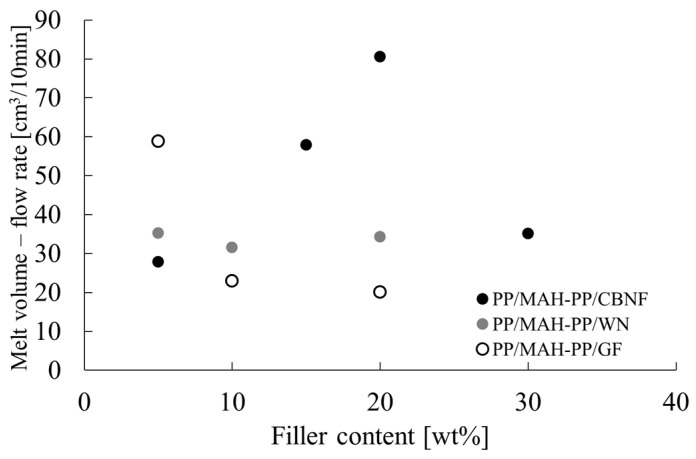
Melt volume–flow rate (MVR) of PP/MAH-PP composites reinforced with carbon nanofiber (CBNF), wollastonite (WN), and glass fiber (GF) as a function of filler content.

Melt flow characteristics are important for evaluating the moldability and processability of composite materials. For this study, melt volume–flow rate (MVR) measurements were taken to investigate the effects of filler type and loading on melt viscosity. Figure 6 portrays the change in MVR relative to filler loading (wt%). MVR is an indicator of the inverse of melt viscosity. A decrease in MVR indicates an increase in melt viscosity (reduced flowability).

The MVR exhibited markedly different behaviors depending on the filler type. PP/MAH-PP/GF showed high MVR of approximately 59 cm^3^/10 min at 5 wt% loading, but the MVR decreased rapidly as the loading increased, reaching approximately 20 cm^3^/10 min at 20 wt%. For PP/MAH-PP/WN, the MVR was approximately 35 cm^3^/10 min at 5 wt%. It remained nearly constant at approximately 34 cm^3^/10 min, even when the loading was increased to 20 wt%. The most unique behavior was observed for PP/MAH-PP/CBNF. The MVR increased from approximately 28 cm^3^/10 min at 5 wt% along with loading, reaching the maximum value recorded for this measurement (approx. 81 cm^3^/10 min) at 20 wt%. However, when loading was increased to 30 wt%, the MVR decreased sharply to approximately 35 cm^3^/10 min.

### 3.3. Flexural Properties of Composites

**Figure 7:**
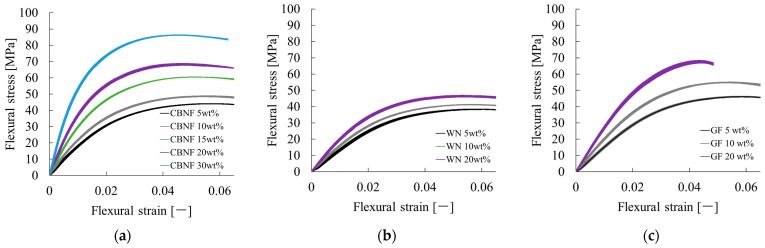
Flexural stress–strain curves of PP/MAH-PP composites reinforced with varying contents of (**a**) CBNF, (**b**) WN, and (**c**) GF. These curves represent the average results of five tests, and the thickness of the lines indicates the degree of variation.

Bending tests and Poisson’s ratio measurements were conducted to evaluate the mechanical properties of the various composites. Figure 7 presents the flexural stress–strain (S-S) curves for composites filled with (a) CBNF, (b) WN, and (c) GF at different weight fractions (5 wt% to 30 wt%).

As the figure shows, a behavior whereby stress increases monotonically with strain was observed for all composites. Linear elastic deformation occurred in the initial stages, followed by a transition to the plastic deformation region after the yield point. Based on this pattern, it was determined that the compositions examined in this study can undergo full-section yielding under flexural loading.

Specifically in the CBNF composites (Figure 7a), marked improvement in strength was confirmed with increasing filler content. Whereas the maximum flexural stress was approximately 45 MPa at 5 wt% addition, it reached approximately 85 MPa at 30 wt%, demonstrating a considerably strong reinforcing effect. In the WN composites (Figure 7b), an increase in stress with higher content was also observed, but the rate of increase was gradual compared to that found for CBNF. The maximum stress at 20 wt% WN remained at approximately 45 MPa, which was clearly lower than that of the CBNF composite at the same concentration (approx. 68 MPa). The GF composite (Figure 7c), used as a reference inorganic filler, exhibited maximum stress of approx. 68 MPa at 20 wt%. This strength level was equivalent to that of the CBNF composite at the same concentration (20 wt%).

**Figure 8:**
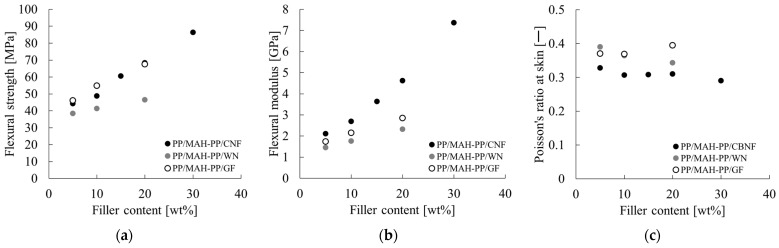
Flexural properties of PP/MAH-PP composites reinforced with carbon nanofiber (CBNF), wollastonite (WN), and glass fiber (GF) as a function of filler content: (**a**) flexural strength, (**b**) flexural modulus, and (**c**) Poisson’s ratio at the skin layer. The standard deviation is indicated by error bars; however, these are too small and consequently difficult to discern.

Figure 8a and Figure 8b, respectively, show changes in flexural strength and flexural modulus relative to filler loading (wt%). Figure 8c shows the change in the Poisson’s ratio of the skin layer relative to filler loading (wt%). Because the standard deviation for each evaluation was less than 1% of the average value, only symbols for the average values are presented in these figures.

As shown in Figure 8a, the flexural strength improved with increasing filler loading in all samples. The PP/MAH-PP/CBNF composite showed the most remarkable strength improvement, reaching approximately 87 MPa at 30 wt% loading. In contrast, the PP/MAH-PP/GF composite was approximately 68 MPa at 20 wt%. The PP/MAH-PP/WN composite was approximately 46 MPa at 20 wt%, confirming that CBNF provides the greatest reinforcing effect. As portrayed in Figure 8b, a similar trend was observed for the flexural modulus. PP/MAH-PP/CBNF showed the steepest increase in modulus relative to loading, achieving a high value of approximately 7.4 GPa at 30 wt%. Also, PP/MAH-PP/GF reached approximately 2.9 GPa at 20 wt%, and PP/MAH-PP/WN reached approximately 2.3 GPa at 20 wt%, collectively demonstrating that the stiffness improvement effect found for CBNF is outstanding. As shown in Figure 8c, the PP/MAH-PP/CBNF composite exhibited the lowest Poisson’s ratio (approx. 0.29–0.33) across all measured loading ranges, with a slight decreasing trend observed as loading increased. In contrast, the values for PP/MAH-PP/GF were approximately 0.37–0.40 and for PP/MAH-PP/WN were approximately 0.34–0.39: both consistently higher than those of the CBNF composite. These results reveal CBNF as an extremely effective reinforcing agent: it improves the strength and stiffness of the PP matrix dramatically compared to conventional inorganic fillers such as GF and WN.

### 3.4. Filler/Matrix Interfacial Adhesion

**Figure 9:**
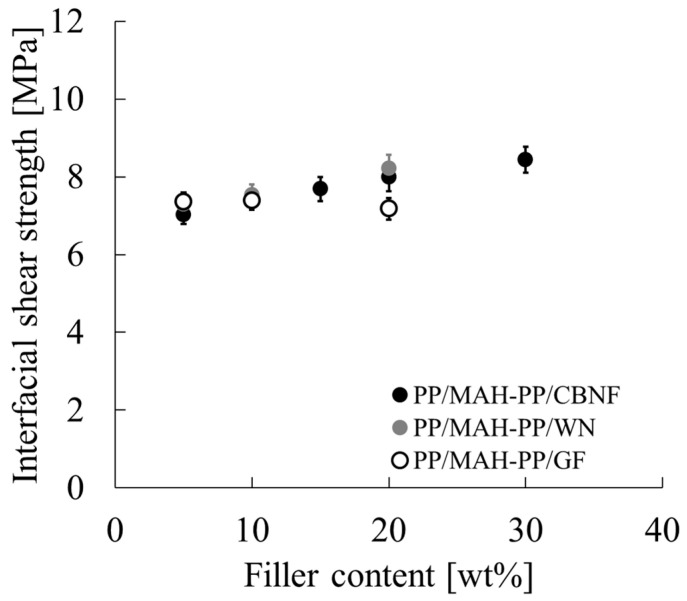
Interfacial shear strength (IFSS) of PP/MAH-PP composites reinforced with carbon nanofiber (CBNF), wollastonite (WN), and glass fiber (GF) as a function of filler content. Error bars represent the standard deviation of the measurements.

To clarify the extent to which the differences in mechanical properties observed in Figure 7 and Figure 8 are attributable to the interfacial adhesion between the matrix and the filler, the interfacial shear strength (IFSS) was evaluated. Figure 9 shows the calculated IFSS results at each filler loading (wt%). Error bars in the figure represent the standard deviation.

The IFSS values for all samples were 7–8.5 MPa. Comparison at 20 wt% loading shows that PP/MAH-PP/GF was approximately 7.2 MPa, whereas PP/MAH-PP/WN was approximately 8.2 MPa and PP/MAH-PP/CBNF was approximately 8.1 MPa. Both CBNF and WN showed higher IFSS values than those found for GF. Furthermore, the CBNF composite showed a trend of gradually increasing IFSS with higher loading, reaching the maximum value of approximately 8.5 MPa at 30 wt%.

### 3.5. Impact Resistance of Composites

**Figure 10:**
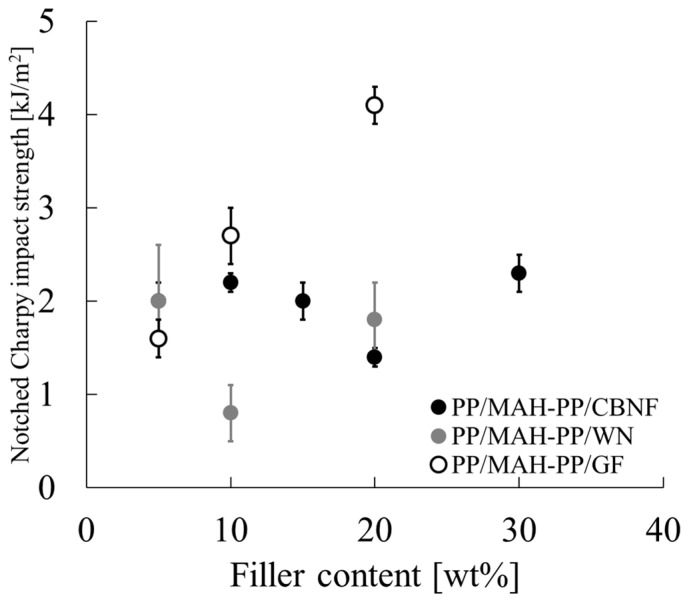
Notched Charpy impact strength of PP/MAH-PP composites reinforced with carbon nanofiber (CBNF), wollastonite (WN), and glass fiber (GF) as a function of filler content. Error bars represent the standard deviation of the measurements.

To evaluate the impact resistance and toughness of the composites, notched Charpy impact tests were conducted. Figure 10 shows the relation between filler loading and Charpy impact strength for the PP/MAH-PP matrix with each filler: CBNF, WN, and GF. The error bars represent the standard deviation.

As Figure 10 clarifies, the impact strength behavior showed completely different trends depending on the filler type. In the GF composite, significant improvement in impact strength was found with increasing filler content. The strength, which was approximately 1.6 kJ/m^2^ at 5 wt%, reached approximately 4.1 kJ/m^2^ at 20 wt%, exhibiting the maximum value found within the scope of this experiment. By contrast, the systems filled with whiskers (CBNF and WN) showed no dramatic improvement with addition seen in GF. The CBNF composite (black circles) remained within 1.4–2.3 kJ/m^2^ across the entire measured concentration range (5–30 wt%). It is noteworthy that, at low concentrations (5 wt%), CBNF (2.0 kJ/m^2^) showed a higher impact value than GF did (1.6 kJ/m^2^). Regarding the WN composite (gray circles), low values or unstable behavior were confirmed compared to other fillers, such as a sharp drop in impact strength to less than 1.0 kJ/m^2^ at 10 wt%.

### 3.6. Fracture Surface Observation

To clarify the failure mechanisms and energy dissipation modes found from the impact tests, the fracture surfaces near the notches of the broken GF and CBNF composite specimens were observed using SEM.

**Figure 11:**
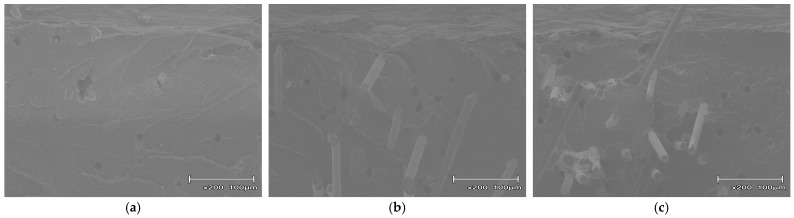
Scanning electron microscopy (SEM) micrographs of the fracture surfaces of PP/MAH-PP/GF composites near the notch tip after Charpy impact testing: (**a**) 5 wt%, (**b**) 10 wt%, and (**c**) 20 wt% GF loading.

Figure 11 shows the fracture surfaces of composites with GF content varied at 5, 10, and 20 wt%. In the fracture surfaces of the GF-based composites, smooth matrix fracture surfaces were observed along with significant fiber pull-out and associated cylindrical voids in all compositions. Particularly in the specimen with 20 wt% GF (Figure 11c), numerous fibers were observed protruding hundreds of micrometers from the fracture surface. Additionally, clear gaps were apparent at the fiber–matrix interface. Almost no matrix resin adhesion was observed on the fiber surfaces.

**Figure 12:**
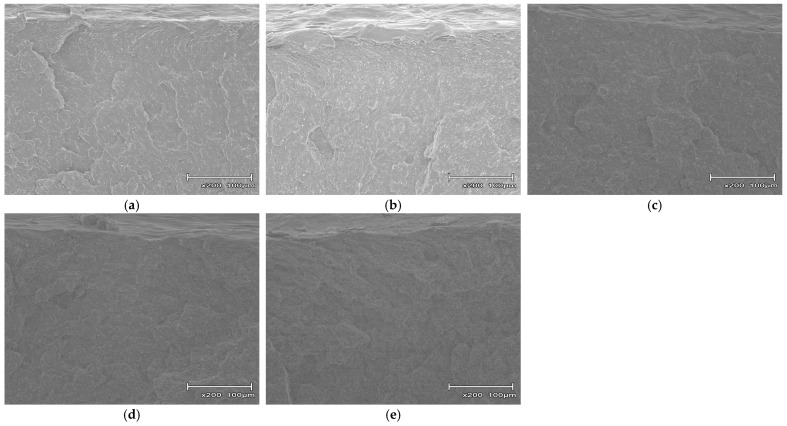
Scanning electron microscopy (SEM) micrographs of the fracture surfaces of PP/MAH-PP/CBNF composites near the notch tip after Charpy impact testing: (**a**) 5 wt%, (**b**) 10 wt%, (**c**) 15 wt%, (**d**) 20 wt%, and (**e**) 30 wt% CBNF loading.

Figure 12 portrays fracture surfaces of composites with CBNF contents varied at 5, 10, 15, 20, and 30 wt%. The fracture surfaces of the CBNF composites exhibited morphology that was completely different from those of the GF-based composites. Specifically, it was difficult to identify the filler at this magnification, and it was also difficult to confirm the presence of aggregates. Because the CBNF content increased from 5 wt% (Figure 12a) to 30 wt% (Figure 12e), it was confirmed that the surface roughness of the fracture surface increased significantly. Particularly in the high loading range of 20–30 wt% (Figure 12d,e), fine irregularities were formed across the entire fracture surface, presenting a complex fracture pattern. Furthermore, macroscopic interfacial debonding and large voids, as seen in the GF-based system, were not observed.

**Figure 13:**
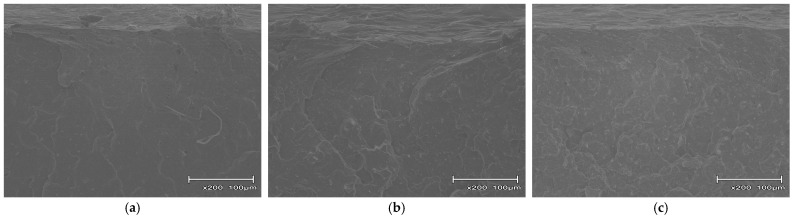
Scanning electron microscopy (SEM) images of the fractured surfaces near the notch tip for WN composites with various filler contents: (**a**) 5 wt%, (**b**) 10 wt%, and (**c**) 20 wt%.

Figure 13 depicts the fracture surfaces of composites with WN content at 5, 10, and 20 wt%. In this system as well, just as in the CBNF system, the filler was uniformly dispersed, and it was difficult to detect any agglomerates. The fracture surfaces of the WN composites resembled those of the CBNF composites (Figure 13) in that they lacked macroscopic defects such as fiber pull-out and voids observed in the GF-based system. This lack of defects suggests that both WN and CBNF form a fine, nanoscale dispersion state.

Summarizing the observations described above, it is clear that failure in GF-based composites is dominated by interfacial debonding between the fiber and matrix followed by fiber pull-out. In contrast, in the CBNF-based and WN-based composites, the fillers are considered to be well-dispersed, forming strong interfaces with the matrix.

## 4. Discussion

### 4.1. Unique Flow Behavior in CBNF Composites

The MVR measurement results presented in Section 3.2 show unique behavior whereby the MVR increased, meaning that flowability improved, up to CBNF loading of 20 wt%. Generally, increasing the filler content in a resin increases viscosity and decreases MVR [33]. The CBNF behavior found from this study runs counter to this general trend. This point is discussed below from the perspective of the CBNF dimensional characteristics and dispersion state.

As described in Section 2.1, CBNF is a whisker-like filler with sub-micrometer lengths and diameters. Consequently, the inter-filler distance in the matrix is considerably shorter than micrometer-order fillers such as WN and GF. This short distance indicates that CBNF exists at a high density even in the immediate vicinity of the mold (or die) wall during the flow field, specifically within the surface skin layer region.

During the flow process, the CBNF dispersed near the surface is exposed at the resin surface, increasing the probability of direct contact with the wall. Because carbon materials possess excellent solid lubricant properties, the exposed CBNF is considered to function as a lubricant against the wall, inducing a “wall slip” effect that reduces the friction coefficient at the interface. This slip effect offset is inferred to surpass the increase in bulk viscosity caused by the added filler, leading to increased apparent MVR.

However, when the loading reached 30 wt%, the MVR began to decrease. This decrease can be interpreted as the structural viscosity (thickening effect) caused by interference between fillers surpassing the flow promotion from the slip effect. This interpretation suggests further that the flowability improvement effect of CBNF has a clear concentration dependence, or threshold.

### 4.2. Yield Conditions for Whisker-Dispersed Composites

Section 3.3 revealed that flexural strength increased monotonically with whisker dispersion. The improvement effect was higher for CBNF than for WN. Furthermore, the reinforcing effect of CBNF on flexural strength was comparable to that of GF when compared on a weight fraction (wt%) basis. However, because CBNF and GF have different densities, the volume fraction (vol%) of filler dispersed in the molded product differs, with CBNF being present in larger amounts. In other words, the flexural strength reinforcement effect per unit volume is highest for GF, followed by CBNF and then WN.

The authors have reported that the improvement in flexural strength caused by the dispersion of micro-sized fibers such as GF is caused primarily by fiber pull-out. The yield initiation stress based on the fiber pull-out model proposed by the authors is determined by the following equation [15].

(8) $$\sigma_{y\text{-}p}=\frac{2\tau_{I}}{\cos\varphi }\frac{l_{p}V_{f}}{dl_{f}}$$

In that equation, τ_I_ denotes the interfacial shear strength, V_f_ is the fiber volume fraction, and φ represents the fiber orientation angle. Additionally, l_p_ represents the pull-out fiber length, l_f_ stands for the remaining fiber length, and d denotes the fiber diameter.

The results shown in Section 3.3 indicate the interfacial shear strength (IFSS) for each composition studied as nearly equivalent. If one assumes that this reinforcement mechanism via fiber pull-out is applicable to whiskers, then WN and CBNF can be predicted to provide a reinforcing effect that is superior to GF because their fiber diameters are smaller and their interfacial specific surface area is larger.

**Figure 14:**
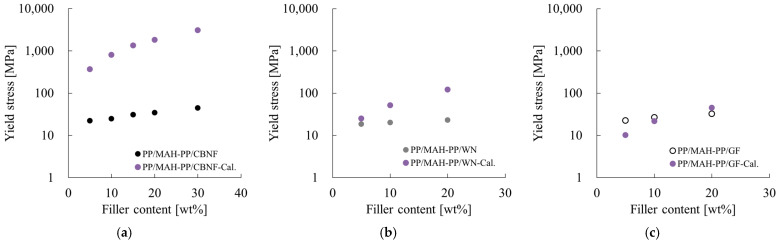
Experimentally obtained yield stress and theoretical values calculated using the pull-out model (Equation (8)) for various composites as a function of filler content: (**a**) PP/MAH-PP/CBNF, (**b**) PP/MAH-PP/WN, and (**c**) PP/MAH-PP/GF.

The yield initiation stress calculated using Equation (8) is presented in Figure 14. Furthermore, the parameters used in Equation (8) are shown in Table 3. Here, the dependence of yield stress on filler loading is shown as a semi-logarithmic graph to verify the suitability of the reinforcement mechanism in each system. The figure also includes the experimentally obtained yield initiation stress results. For the calculation, it was assumed that the whiskers are dispersed without breaking during compounding and that they are perfectly oriented parallel to the flow direction. Furthermore, the pull-out length was assumed to be shorter than the critical fiber length, calculated as half of the remaining fiber length. The values determined under these orientation assumptions correspond to the minimum yield initiation stress according to Equation (8). If the experimentally obtained values fall below these calculated values, then yielding attributable to pull-out cannot occur in that composition.

First, particularly addressing the CBNF-filled system (Figure 14a) and the WN-filled system (Figure 14b), a large discrepancy was observed between the experimentally obtained and calculated values in both systems. Although the calculated values predicted an exponential surge with increasing filler loading, the experimentally obtained values showed only a slight increasing trend. Particularly in the high-loading region, the experimentally obtained values were several orders of magnitude lower than the calculated values. In the GF-filled system (Figure 14c), the experimentally obtained and calculated values showed good agreement or a trend where the former exceeded the latter in the region where loading was 10 wt% or less. However, upon reaching the high-loading region of 20 wt%, the experimentally obtained values fell below the calculated values; a divergence in the increase behavior was observed.

The relation between the experimentally obtained and calculated values in these results suggests important implications for identifying the yield mechanism inside the composite. Generally for this type of theoretical model, if the experimentally obtained values are lower than the calculated values, then it is inferred that the assumed stress transfer mechanism is not fully functioning. In the CBNF and WN systems, where the experimentally obtained results were markedly less than the calculated results, yielding caused by filler “pull-out” can be regarded as not theoretically dominant. This inferred lack of dominance is true probably because other failure modes, such as local plastic deformation of the matrix, became dominant before pull-out can occur at the interface between the fine filler and the matrix. Similarly, in the GF system, the fact that experimentally obtained values fell below the calculated values at 20 wt% indicates that yielding caused by pull-out did not occur in this region. Regarding this discrepancy at 20 wt% GF, the increase in stress concentration is reportedly associated with high loading caused the adhesion strength at the filler–matrix interface to reach its limit. Consequently, “interfacial debonding” before pull-out occurred [15].

From the discussion presented above, yield conditions when fine fillers such as whiskers are dispersed are considered to be governed by the matrix behavior rather than by filler pull-out. Based on this assumption, we discuss the matrix using a shear yielding model. The authors proposed the following Equation (9), assuming that molecular friction results from thermal strain acting inside thermoplastic injection molded products, thereby inducing shear yielding of the entire system [34].

(9) $$\sigma_{y\text{-}s}=C\alpha \Delta TE\cos\theta$$

In that equation, υ stands for Poisson’s ratio, ΔT represents the difference between the molding temperature and the test temperature, and E denotes the Young’s modulus. C is a coefficient: the square root of 3 for PP following the Mises yield criterion [35]. Furthermore, θ is the shear angle, expressed by the following Equation (10) [34].

(10) $$\theta =\tan^{-1}2\upsilon$$

**Figure 15:**
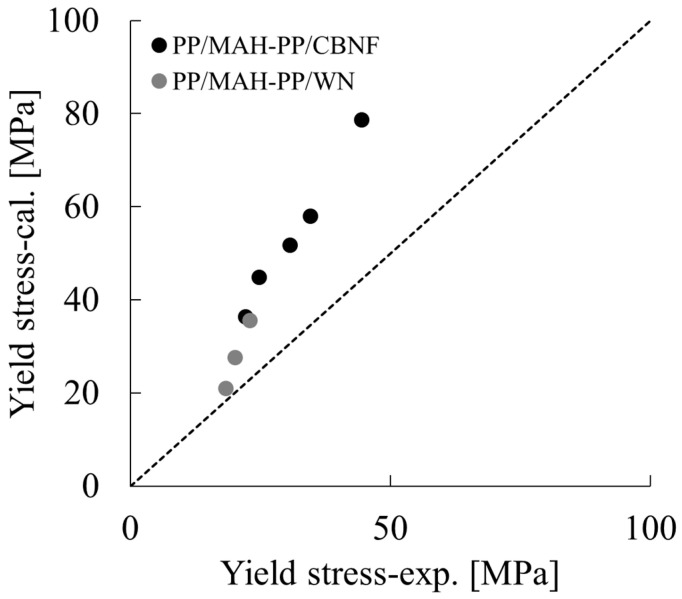
Correlation plot between the experimentally obtained yield stress (σ_y-exp._) and the theoretical yield stress calculated using the molecular friction model (σ_y-s_) for PP/MAH-PP/CBNF (black circles) and PP/MAH-PP/WN (grey circles) composites. The dashed line represents the ideal 1:1 correlation (σ_y-exp._ = σ_y-s._).

It is noteworthy that E was determined from the following Equation (11).

(11) $$E=\frac{\left(1-2\upsilon \right)\left(1+\upsilon \right)}{1-\upsilon }E_{f}$$

where E_f_ means the flexural modulus. Figure 15 presents the results of comparing the yield initiation stress determined using Equations (9)–(11) with the experimentally obtained values. The dashed line in the figure represents the ideal state where the experimentally obtained and calculated values match perfectly.

As Figure 15 clarifies, positive correlation was confirmed between the experimentally obtained and calculated values in both the PP/MAH-PP/WN and PP/MAH-PP/CBNF systems. Specifically, the experimentally obtained yield stress consistently showed values lower than the theoretical calculated values. Particularly in the PP/MAH-PP/CBNF system, the deviation (difference between experimentally obtained and calculated values) was considerable; under conditions in which the experimentally obtained value was approximately 45 MPa, the calculated value reached approximately 80 MPa. By contrast, although the deviation in the PP/MAH-PP/WN system was smaller than the CBNF system, it similarly exhibited a positive deviation.

The behavior by which experimentally obtained values fall markedly below theoretical predictions suggests contributions by reinforcement mechanisms within the actual composite material that are insufficiently reflected in Equation (9). Generally, when experimentally obtained values fall below theoretical values, poor interfacial adhesion is suspected.

Results of this study suggest that external stress arising from this interaction acted on the shear plane separately from thermal strain, thereby promoting molecular friction (sliding). Consequently, Equation (9) was modified to the following Equation (12).

(12) $$\sigma_{y\text{-}s}^{\prime }=C\cos\theta \left(\alpha \Delta TE+\sigma_{i}\right)$$

**Figure 16:**
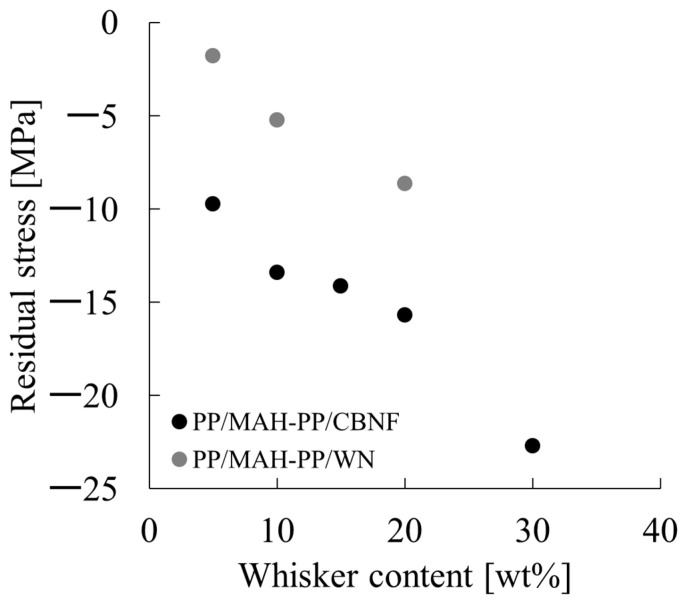
Residual stress as a function of whisker content for PP/MAH-PP/CBNF (black circles) and PP/MAH-PP/WN (grey circles) composites. Residual stress was calculated as the difference between the experimentally obtained yield stress and the theoretical value predicted by the molecular friction model (σ_y-exp._–σ_y-s_).

Therein, σ_i_ represents the external stress (constraint stress) occurring in the matrix. The results of determining this value are presented in Figure 16. From these results, behavior by which σ_i_ decreased monotonically with increasing whisker content was observed for both PP/MAH-PP/CBNF and PP/MAH-PP/WN. Specifically, it reached approximately −10 MPa at 5 wt% and −23 MPa at 30 wt% in the PP/MAH-PP/CBNF system, whereas it remained at approximately −2 MPa at 5 wt% and −9 MPa at 20 wt% in the PP/MAH-PP/WN system. When compared at the same whisker content, the CBNF system consistently showed markedly lower values than the WN system.

In this measurement, a negative value for σ_i_ indicates that residual stress in the expansion direction is acting on the matrix resin. The generation and increase in such residual stress are interpreted as originating from interfacial interaction occurring between the matrix and the filler: the deformation and thermal shrinkage of the matrix occurring during the molding process are constrained by interaction at the interface with the filler, causing stress to accumulate within the system. Therefore, the fact that higher expansive residual stress developed in the CBNF system than in the WN system suggests that the interfacial interaction with the matrix is stronger for CBNF. As a result, the constraint effect on the matrix works more efficiently. The primary cause for this is likely the size effect of the filler. Because the fiber diameter of CBNF is about one order of magnitude smaller than that of WN, the specific surface area increases significantly. However, the distance between fillers becomes closer. The interfacial interaction force decreases. The authors have reported that the residual stress attributable to interfacial interaction is proportional to the product of the interfacial interaction force and the specific surface area [36]. Because the increase in specific surface area is more dominant than the decrease in interfacial interaction force caused by the closer inter-filler distance, it is considered that CBNF produced larger residual stress. To supplement this hypothesis, we calculated the distance between fillers, <L>, using the following Equation (13) and determined its relationship with σ_i_, as calculated in this paper [37].

(13) $$<L>=D\left[\sqrt[{3}]{\frac{\pi }{6V_{f}}}-1\right]$$

Here, D is the equivalent particle diameter calculated using the following Equation (14) [37].

(14) $$D=\sqrt[{3}]{\frac{3}{2}d_{f}^{2}L_{f}}$$

**Figure 17:**
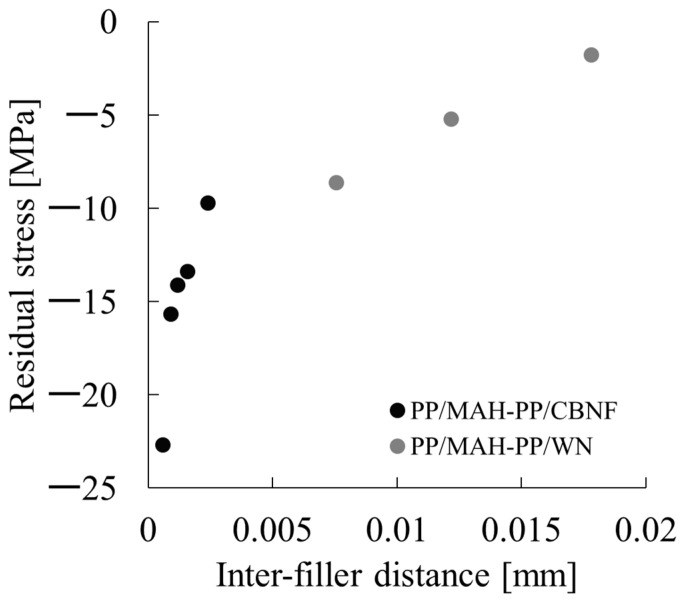
Scatter plot showing the relationship between inter-filler distance and residual stress in PP/MAH-PP/CBNF and PP/MAH-PP/WN composites.

Here, d_f_ denotes the fiber diameter and L_f_ denotes the fiber length. The relationship between the inter-filler distance calculated from the above equation and σ_i_ is shown in Figure 17. As is evident from the figure, in both the PP/MAH-PP/CBNF and PP/MAH-PP/WN composite systems, the residual stress exhibited larger negative values (compressive stress) as the inter-filler distance decreased. For the PP/MAH-PP/CBNF composite, the data is concentrated in the region of very narrow filler-to-filler distances (less than 0.005 mm), and the residual stress in this region shifted significantly in the negative direction, ranging from approximately −10 MPa to −23 MPa. In contrast, the PP/MAH-PP/WN composite exhibited a relatively wide range of filler-to-filler distances from 0.007 mm to 0.018 mm, and the residual stress remained within the range of approximately −2 MPa to −9 MPa. Comparing the two, it is presumed that the composite material using CBNF had a significantly shorter inter-filler distance than the system using WN, and consequently, the compressive residual stress generated was significantly larger.

The discussion presented above and subsequent verification clarify that the yield conditions for whisker-dispersed composites are explainable not by filler pull-out, but by a matrix shear yielding model that accounts for interfacial constraint.

### 4.3. Impact Energy Dissipation Mechanism of Whisker-Dispersed Composites

As stated in Section 3.4, no improvement in notched impact strength was observed in whisker-dispersed systems. The primary cause is probably the difference in failure mode discussed in Section 3.5. This inference suggests that the energy absorption mechanism that contributed to the GF-dispersed system is not functioning effectively in the whisker-dispersed systems. Hereinafter, we examine the impact energy dissipation mechanism of whisker-dispersed composites, combining the findings with the yield mechanism discussed in Section 4.2.

In Section 4.2, we concluded that the yield conditions of whisker-dispersed composites are governed by the shear yielding of the matrix considering interfacial constraint, rather than filler pull-out. Based on the results presented in Section 3.5, we assume that a similar mechanism applies to deformation in high strain rate regions such as the notch tip. Then we derive the critical energy release rate using the crack tip opening displacement, which is a linear elastic fracture mechanics parameter, to verify its validity.

When plastic deformation in the crack tip region cannot be ignored, the critical energy release rate G_c_ is expressed by the following Equation (15) [38].

(15) $$G_{c}=\frac{\pi^{2}\kappa \sigma_{y}\delta }{8\ln\left(\sec\frac{\pi \sigma }{2\kappa \sigma_{y}}\right)}$$

In that equation, δ denotes the crack tip opening displacement, κ represents the plastic constraint factor, σ_y_ represents the yield initiation stress, and σ expresses the stress in the unconstrained region. Furthermore, according to the Dugdale model, the crack tip opening displacement δ is given by the following Equation (16) [38].

(16) $$\delta =\frac{8a\kappa \sigma_{y}\left(1-\upsilon^{2}\right)}{\pi E}\ln\left(\sec\frac{\pi \sigma }{2{\kappa \sigma }_{y}}\right)$$

Therein, a denotes the crack length. According to Williams et al., the Charpy impact strength found by notched impact testing is quantified using Equation (17) [39] as

(17) $$G_{c}=\frac{a_{iN}}{\Phi }\left(1-\frac{a}{w}\right),$$

where w is the specimen width. Also, Φ is a geometry factor, calculated using the following Equation (18) for Charpy impact tests.

(18) $$\Phi =\frac{a}{2w}+\frac{1}{18\pi }\frac{S}{w}\frac{1}{\left(a/w\right)}$$

In that equation, S is the support span. For this study, we calculated G_c_ using the theory presented by Williams et al. and compared it with G_c_ as found from Equations (15) and (16). If both show values are of the same order, then it can be inferred that the impact energy dissipation mechanism of whisker-dispersed composites is the shear yielding of the matrix considering interfacial constraint.

**Figure 18:**
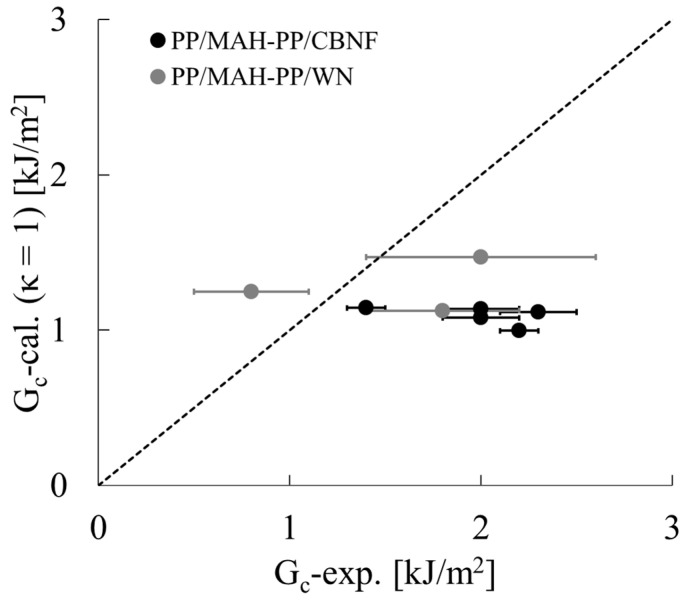
Comparison between experimentally obtained fracture toughness (G_c_-exp.) and calculated fracture toughness (G_c_-cal.) for PP/MAH-PP/CBNF and PP/MAH-PP/WN composites. Error bars represent the standard deviation of the measurements. Calculated values were obtained using a theoretical model with a correction factor set as κ = 1. The dashed line represents the identity line (y = x), signifying the ideal correlation by which experimentally obtained results match the theoretical predictions.

The verification results are shown in Figure 18. It is noteworthy that G_c_-cal. was calculated assuming a geometric factor κ = 1. The dashed line in the figure represents the theoretical line at which experimentally obtained and calculated values match. However, for both PP/MAH-PP/CBNF and PP/MAH-PP/WN, the data points are distributed overall in the region below the dashed line: the experimentally obtained values (G_c_-exp.) were higher than the theoretical calculated values (G_c_-cal.) for all samples. Particularly, the discrepancy between experimentally obtained and calculated values became large in the region where G_c_-exp. exceeded 1.5 kJ/m^2^. Although some WN samples (near G_c_-exp. approx. 0.8 kJ/m^2^) showed behavior close to the dashed line, the increase in calculated values did not follow the increase in experimentally obtained values as the system moved toward the high-toughness side.

This result suggests that the theoretical model assuming κ = 1 underestimates the fracture energy in actual composites. The fact that experimentally obtained values exceed calculated values means that energy dissipation attributable to shear deformation of the matrix near the crack tip is progressing in the actual fracture process. The influence of the plastic constraint occurring there cannot be ignored. Therefore, for accurate description of the reinforcement mechanism in this system, the κ value must be re-evaluated as a value greater than 1, appropriately reflecting interfacial interaction.

Furthermore, the conditions under which the material properties used in the calculations were obtained are also believed to contribute to the discrepancy between the calculated and experimental values. The material properties used in this calculation were obtained from quasi-static tests, which differ from the loading rates encountered in impact tests. Considering the viscoelasticity of polymeric materials, it is expected that the elastic modulus and yield stress would be higher at the high loading rates encountered in impact tests than at quasi-static loading rates. Therefore, it is inferred that the reason the calculated results yielded lower values than the experimental results stems not only from the aforementioned factor that κ is not equal to 1, but also from the fact that the elastic modulus and yield stress could not be evaluated at the same loading rates as those in the impact test. Considering the loading rate dependence in this regard and conducting a detailed verification are topics for future research.

The discussion presented above clarifies that the impact energy dissipation mechanism of whisker-dispersed composites is driven primarily by the shear deformation of the matrix rather than filler pull-out, and can be reasonably explained by considering the plastic constraint near the crack tip. Although this consideration indicates that it is difficult to improve notched impact strength simply by dispersing whiskers in the same way as GF, it also suggests application of methods to improve the toughness of the matrix polymer itself.

Based on this guideline, a future prospect is to introduce elastomers that contribute to the high toughening of the matrix and to investigate the mechanical properties in a co-dispersed system with whiskers. Because elastomer particles induce local plastic deformation of the matrix as stress concentration sources, a synergistic effect with the “shear-deformation-led” energy dissipation mechanism revealed in this study is expected. Therefore, we plan to proceed with detailed elucidation of the interfacial interactions among the three phases and their effects on impact properties when whiskers (responsible for stiffness) and elastomers (responsible for toughness) are microscopically controlled and dispersed.

## 5. Conclusions

This study evaluated the mechanical properties of composite materials consisting of isotactic polypropylene (iPP) and whisker-like fillers, specifically carbon nanofibers (CBNF) and wollastonite (WN). The underlying reinforcement mechanisms were investigated through comparative analysis conducted with conventional glass fiber (GF) reinforced materials.

Regarding the elastic modulus, effective improvements were confirmed with the addition of both CBNF and WN. The CBNF composite exhibited a high flexural modulus of approximately 7.4 GPa at 30 wt% loading. This behavior is explainable by the conventional rule of mixtures, demonstrating that the high stiffness of the fillers themselves contributes directly to the overall rigidity of the composite.

By contrast, properties in the large-deformation region, such as yield stress and fracture toughness, exhibited behaviors different from the elastic modulus. Fiber pull-out, which serves as the primary energy dissipation mechanism in GF-reinforced systems, was not dominant in the whisker-based systems used for this study: experimentally obtained values fell below the pull-out model predictions. Detailed analysis revealed that these properties are governed by a “matrix-dominated” mechanism that depends strongly on the shear yielding behavior of the iPP matrix, which indicates that the plastic deformation capacity of the matrix is the primary factor determining the fracture properties of these composites.

Additionally, unique flow behavior was observed in the CBNF-filled system, where the melt volume–flow rate (MVR) increased until the loading reached 20 wt%. This increase is attributed to the fine CBNF inducing a wall slip effect near the mold surfaces during the flow process.

These findings indicate the necessity of considering the inherent ductility and shear yield characteristics of the matrix material when designing high-performance composites using whisker-like fillers. Moreover, it is necessary to control the filler type and dispersion state. The mechanical model developed for this study enables the quantitative evaluation of these contributions and provides useful guidelines for the development of next-generation composite materials with high recyclability.

## Figures and Tables

**Table 1:** Characteristics of the raw materials used in this study, including their classification, product names, manufacturers, and physical specifications.

Category	Material Name (Abbreviation)	Product Name/Manufacturer	Specifications/Notes
Matrix	Isotactic polypropylene (iPP)	Novatec PP MA1B/Japan Polypropylene Corp., Tokyo, Japan	—
Coupling Agent	Maleic anhydride-modified polypropylene (MAHPP)	SCONA TSPP 10213 GB/BYK Additives & Instruments Co., Ltd., Wesel, Germany	Used to improve interfacial adhesion
Filler	Carbon nanofibers (CBNF)	ALP-NA1/Almedio Inc., Tokyo, Japan	Diameter: 0.2–0.8 µm Length: 1–15 µm
Filler	Wollastonite (WN)	WFB5/Nippon Talc Co., Ltd., Osaka, Japan	Diameter: 5–6 µm Avg. Length: 60–72 µm
Filler	Glass fibers (GF)	T-351/Nippon Electric Glass Co., Ltd., Shiga, Japan	Diameter: 13 µm Initial Length: 3 mm

**Table 2:** Injection molding conditions.

PP [wt%]	MAH-PP [wt%]	CBNF [wt%]	WN [wt%]	GF [wt%]	T_inj_ [°C]	T_mold_ [°C]	V_inj_ [mm/s]	P_hold_ [MPa]	T_inj_ [s]	T_cool_ [s]
92	3	5	-	-	230	50	30	70	15	20
87	3	10	-	-	230	50	30	70	15	20
80.5	4.5	15	-	-	230	50	30	56	15	20
74	6	20	-	-	230	50	30	56	15	20
61	9	30	-	-	240	80	30	56	15	20
92	3	-	5	-	230	50	30	42	15	20
87	3	-	10	-	230	50	30	56	15	20
74	6	-	20	-	230	50	30	63	15	20
92	3	-	-	5	230	50	30	42	15	20
87	3	-	-	10	230	50	30	70	15	20
74	6	-	-	20	230	50	30	70	15	20

**Table 3:** Theoretical yield initiation stress (σ_y-p_) calculated from the fiber pull-out model and experimentally obtained yield initiation stress (σ_y_) obtained from three-point bending tests for PP/MAH-PP composites reinforced with CBNF, WN, and GF.

PP [wt%]	MAH-PP [wt%]	CBNF [wt%]	WN [wt%]	GF [wt%]	V_f_ [vol%]	τ_I_ [MPa]	d [μm]	σ_y-p_ [MPa]	σ_y_ [MPa]
92	3	5	-	-	2.6	7.0	0.5	365	22
87	3	10	-	-	5.4	7.4	0.5	804	25
80.5	4.5	15	-	-	8.3	8.1	0.5	1341	31
74	6	20	-	-	11.4	8.0	0.5	1822	35
61	9	30	-	-	18.1	8.4	0.5	3055	45
92	3	-	5	-	1.7	7.4	5	25	18
87	3	-	10	-	3.4	7.5	5	51	20
74	6	-	20	-	7.4	8.2	5	122	23
92	3	-	-	5	1.8	7.4	13	10	22
87	3	-	-	10	3.8	7.4	13	22	27
74	6	-	-	20	8.1	7.2	13	45	32

## Data Availability

The original contributions described as a result of this study are included in the article. Further inquiries can be directed to the corresponding author.
